# Ecophysiology of *Fusarium chaquense* a Novel Type A Trichothecene Producer Species Isolated from Natural Grasses

**DOI:** 10.3390/toxins13120895

**Published:** 2021-12-13

**Authors:** Maria J. Nichea, Eugenia Cendoya, Miriam Haidukowski, Adriana M. Torres, María L. Ramirez

**Affiliations:** 1 Research Institute on Mycology and Mycotoxicology (IMICO), National Scientific and Technical Research Council-National University of Río Cuarto (CONICET-UNRC), Río Cuarto 5800, Argentina; mnichea@exa.unrc.edu.ar (M.J.N.); ecendoya@exa.unrc.edu.ar (E.C.); atorres@exa.unrc.edu.ar (A.M.T.); 2 Institute of Sciences of Food Production, CNR, 70126 Bari, Italy; miriam.haidukowski@ispa.cnr.it

**Keywords:** *Fusarium chaquense*, Poaceae, ecophysiology, beauvericin, trichothecenes

## Abstract

*Fusarium chaquense*, a recently formally described novel species, has been identified as an T-2 toxin (T-2), HT-2 toxin (HT-2) and other toxins producer in natural grasses (Poaceae) from Argentina. The major objective of this study was to describe the effect of water activity (a_W_, 0.995, 0.98, 0.95, 0.93 and 0.91), temperature (15, 25 and 30 °C) and incubation time (5, 15 and 25 days) on growth and to evaluate the production of T-2, HT-2 toxins and beauvericin (BEA) by two *F. chaquense* strains in a grass-based media. The results showed a wide range of conditions for *F. chaquense* growth and mycotoxin production. Both strains had a maximum growth rate at the highest a_W_ (0.995) and 25 °C. Regarding mycotoxin production, more T-2 than the other analysed mycotoxins were produced by the two strains. T-2 production was favoured at 0.995 a_W_ and 30 °C, while HT-2 production at 0.98–0.95 a_W_ and 15 °C. The maximum levels of BEA were produced at 0.995 a_W_ and 25–30 °C. Two-dimensional profiles of a_W_ by temperature interactions were obtained from these data in order to identify areas where conditions indicate a significant risk of mycotoxins accumulation on grass. For its versatility on growth and mycotoxin production in a wide range of a_W_ and temperatures, *F. chaquense* would have an adaptive advantage over other *Fusarium* species, and this would explain its high frequency of isolation in natural grasses grown up in the Chaco wetlands.

## 1. Introduction

*Fusarium chaquense* is a recently described species isolated from asymptomatic native grasses (Poaceae) from a wetland ecosystem of the Chaco Province, Argentina, intended for grazing cattle. DNA sequence-based phylogenetic analyses indicated that *F. chaquense* is closely related to *F. armeniacum* and nested within a clade of primarily type A trichothecene-producing species of the *Fusarium sambucinum* species complex (FSAMSC) [1]. This clade included *F. armeniacum*, *F. langsethiae*, *F. sibiricum*, *F. sporotrichioides*, *F. palustre* and *F. goolgardi*, which are among the few species that produce type A trichothecenes that have an acyl (e.g., T-2 toxin) or hydroxyl (e.g., neosolaniol) group at C8 and no oxygen atom at C7 [2,3]. The analysis of genome sequences also revealed that *F. chaquense* has genes required for trichothecene biosynthesis. In chemical analysis, *F. chaquense* isolates produced the type A trichothecenes T-2 toxin (T-2) and HT-2 toxin (HT-2), neosolaniol (NEO), diacetoxyscirpenol (DAS) and monoacetoxyscirpenol (MAS), as well as the mycotoxin beauvericin (BEA) and the pigment aurofusarin (AUF). None of the *F. chaquense* isolates examined produced any known type B trichothecenes [1]. These novel species were recovered as part of a previous analysis of 175 grass samples representative of 12 genera of the family Poaceae from an area known as Chaco Wetlands (Ramsar site no. 1366, 27°20’ S 058°50’ W) intended to graze cattle, during 2011 and 2014. All the samples were contaminated with mycotoxins, including zearalenone (ZEA), which was present at up to 2000 µg/kg, and the type A trichothecenes T-2 and HT-2, which were each present at up to 5000 µg/kg [4]. Other fungal metabolites detected in the grass samples included the mycotoxins BEA and equisetin and the pigment AUF [4]. Mycological analysis revealed that 100% of sampled plants were infected with *Fusarium*, with *F. chaquense* being the most common species found (61%). These findings indicate that *F. chaquense* is a likely source of observed contamination of Chaco wetland grasses with type A trichothecenes [4]. In contrast, the *F. chaquense* isolates examined by Nichea et al. [1] did not produce the mycotoxin ZEA, and they did not have ZEA biosynthetic genes in their genome sequences. Therefore, the contamination of Chaco wetland grasses with ZEA, as reported by Nichea et al. [1], was almost certainly caused by another fungal species.

Recently Laraba et al. [5] conducted a study about the diversity and trichothecene potential of selected strains belonging to the FSAMSC in order to analyse the phylogenetic diversity of this species complex. Nichea et al. [1] compared partial *RPB2* and *TEF1* sequences of some *F. chaquense* strains with the aforementioned study and indicated that they are 100% identical to another phylogenetically but not formally described species, *Fusarium* sp. nov.-1. This indicates that *F. chaquense* and *Fusarium* sp. nov.-1 are conspecific (*Fusarium* sp. nov.-1 is hereafter referred to as *F. chaquense*). One of the two *F. chaquense* isolates examined by Laraba et al. [5] was recovered from the soil in Australia, and the other was from oats in South Africa. The isolation of *F. chaquense* from sources in Argentina, Australia and South Africa indicates that the species has a relatively wide distribution in the Southern Hemisphere [1].

Trichothecenes represent one of the major classes of mycotoxins, and they cause a significant economic impact on cereal crops each year [6,7]. Over 200 trichothecene analogs have been identified, all of which share the tricyclic 12,13-epoxytrichothec-9-ene (EPT) core structure [8]. These analogs were classified into four groups (Types A, B, C and D) based on the substitution pattern of EPT. Based on the substitution at the C-8 position, types A, B and C trichothecenes can be differentiated [9]. Type A trichothecenes include compounds that have a hydroxyl group at C-8 (e.g., NEO), an ester function at C-8 (e.g., T-2) or no oxygen substitution at C-8 (e.g., DAS). Type A trichothecenes are highly toxic to animals; they cause immune disorders, weight loss, growth retardation, pathological changes in liver cells and death. Moreover, these toxins can inhibit mitosis, nucleic acids and proteins synthesis, as well as induce apoptosis. In plants, DAS and T-2 toxins can cause chlorosis and inhibit coleoptile and root elongation [6].

It is well known that fungal growth and mycotoxin production are a result of the complex interaction of several factors; thus, understanding each factor is essential to understand the overall process to predict and prevent mycotoxin development [10]. Water activity (a_W_) and temperature are the primary environmental factors that influence growth and mycotoxin production by several *Fusarium* species [11].

Scarce information about the ecology of *Fusarium* species in natural ecosystems is available, although surveys in the USA and Australia suggest that *Fusarium* species are commonly found in poaceas in grassland ecosystems [12,13,14]. Moreover, it is important to remark that *F. chaquense* was also isolated from oats in South Africa, with this cereal being susceptible to trichothecene contamination. As part of *F. chaquense* characterisation, we consider it important to study the impact of water activity (a_W_) and temperature on growth and mycotoxins production (T-2, HT-2 and BEA) by two strains of *F. chaquense* isolated from natural grasses. The results obtained from this type of study allow the identification of climate conditions that could indicate a significant risk of mycotoxin accumulation on grasses. As *F. chaquense* is associated with natural grasses in Argentina intended for cattle consumption, their contamination with mycotoxins should not be underestimated in order to preserve animal health.

## 2. Results

### 2.1. Effect of Water Activity and Temperature on Lag Phase and Growth

**Figure 1:**
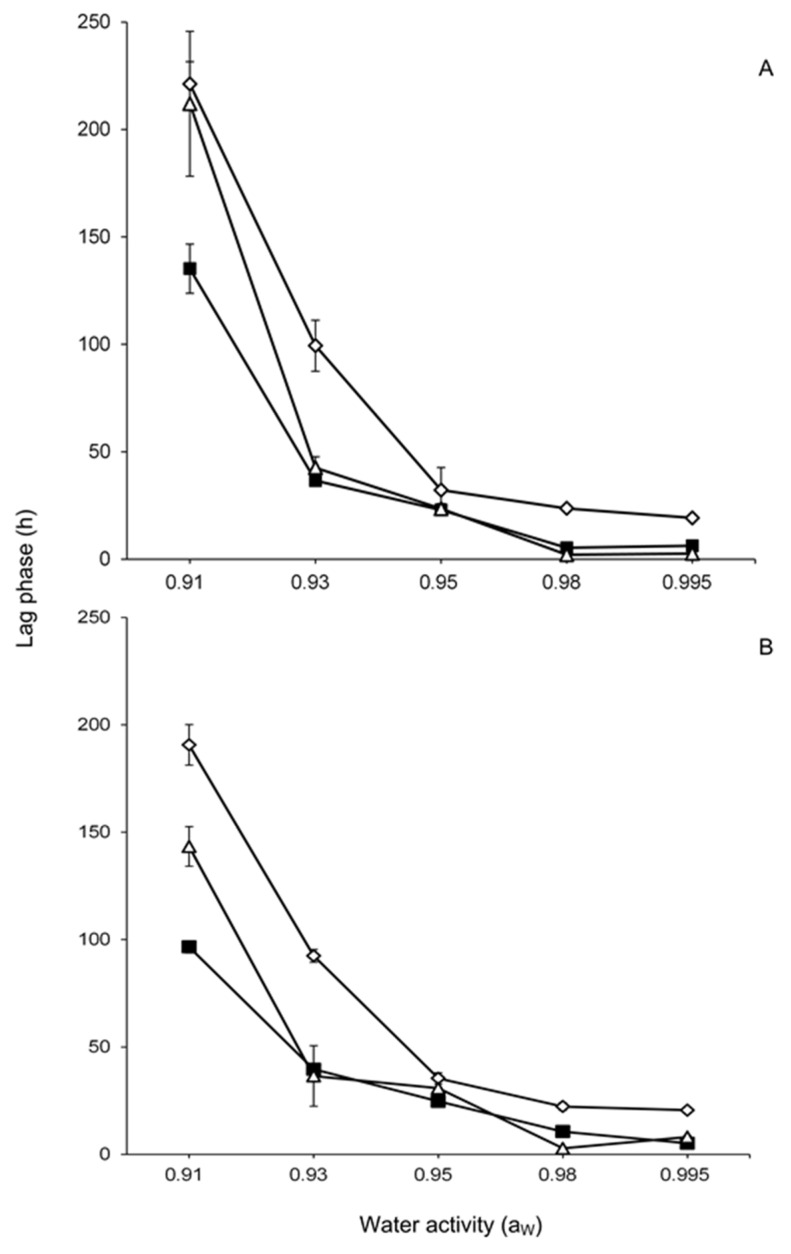
Effect of water activity (0.91–0.995) and temperature 15 °C (◊), 25 °C (■), 30 °C (△) on lag phase of two *Fusarium chaquense* strains in the grass-based culture media (**A**: NRRL 66748; **B**: NRRL 66749). The error bars represent the standard deviation for the triplicates.

The effect of temperature and a_W_ on *F. chaquense* lag phase is shown in Figure 1. In general, both strains showed similar behaviour. The lag phase increased when temperature and a_W_ decreased. Lag phases were shorter (<24 h) at 0.995 and 0.98 at all temperatures analysed. Lag phases increased considerably at the lowest temperature studied (15 °C) and at low a_W_ levels.

**Figure 2:**
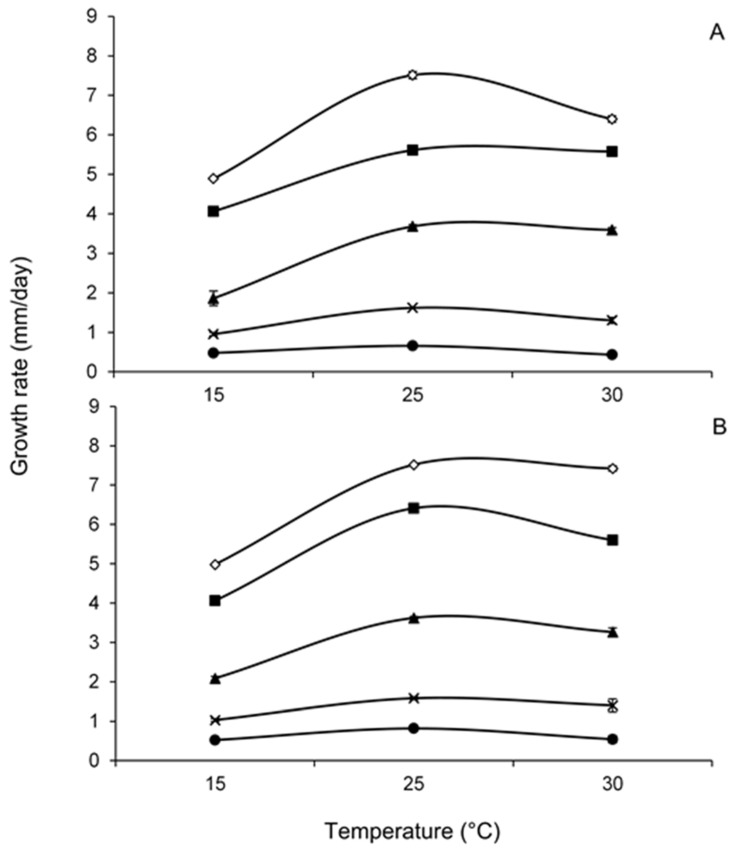
Effect of water activity, 0.91 (●), 0.93 (X), 0.95 (▲), 0.98 (■), 0.995 (◊) and temperature on growth rate of two *Fusarium chaquense* strains in the grass-based culture (**A**: NRRL 66748; **B**: NRRL 66749). The error bars represent the standard deviation for the triplicates.

Figure 2 compares the interaction between a_W_ and temperature on growth rates of two *F. chaquense* strains grown in a grass-based media. Both strains showed similar behaviour, and they were able to grow at a_W_ levels ranging from 0.995 to 0.91 at 15–30 °C with maximum growth rates obtained at the highest a_W_ (0.995) and 25 °C. Growth of both strains decreased as a_W_ of the media was reduced. Regarding incubation temperature, the highest growth rates were obtained at 25 °C, decreasing in the following order: 30 and 15 °C, regardless of a_W_. The two strains were able to grow at the lowest a_W_ tested (0.91) at the three assayed temperatures.

The analysis of variance separately performed in order to analyse the effect of the single variables considered in the study (strain, a_W_, temperature) and two- and three-way interactions revealed that all variables alone and all interactions had a significant effect on lag phases and growth rates. The most significant variable was a_W_ for lag phases and strain x a_W_ interaction for growth rates (Table 1).

**Figure 3:**
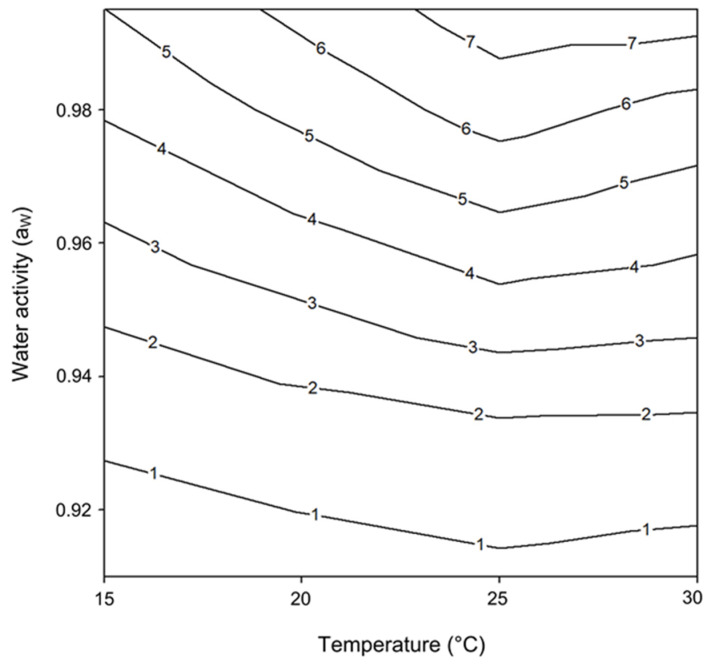
Two-dimensional contour map of *Fusarium chaquense* growth profile in relation to temperature and water activity. The numbers on the isopleths refer to similar growth rates (mm/day).

Contour lines to map the relative optimum and marginal conditions that allowed *F. chaquense* growth were performed (Figure 3). These contour maps show that the growth rate ranged from 0.995 to 0.95 a_W_ at 25–30 °C, indicating possible optimum conditions for growth for both *F. chaquense* strains.

### 2.2. Effect of a_W_, Temperature and Incubation Time on Mycotoxin Production

**Figure 4:**
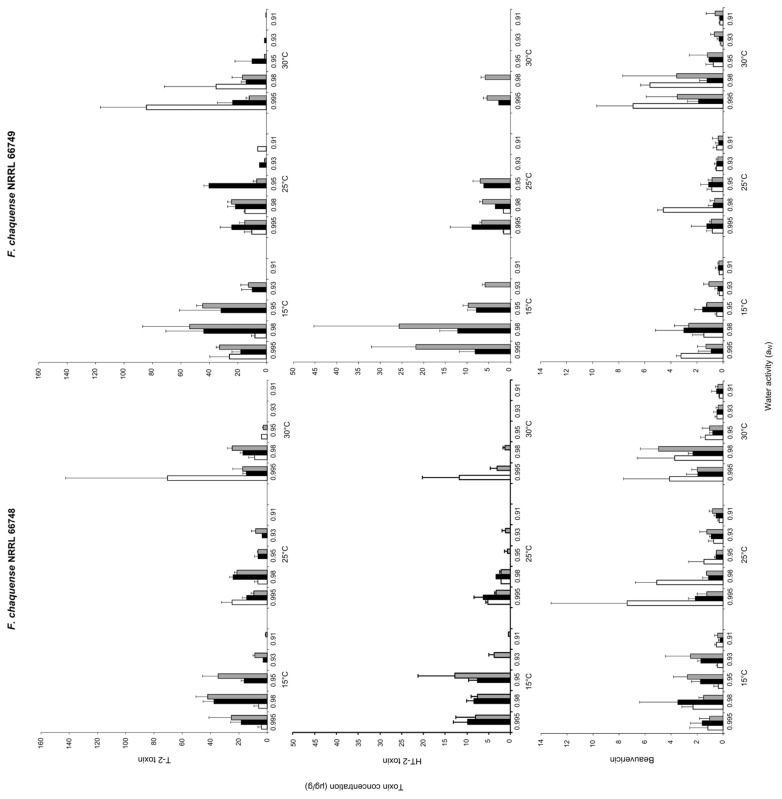
Toxins concentrations T-2, HT-2 and BEA (μg/g) produced by *Fusarium chaquense* strains NRRL 66748 and NRRL 66749 in the grass-based culture adjusted to different a_W_ levels and temperatures at different incubated times. 5 (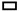), 15 (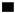) and 25 (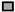) days.

Figure 4 shows the effect of a_W_, temperature and incubation time on T-2, HT-2 and BEA production by both strains of *F. chaquense* grown in a grass-based media over 25 days.

Maximum T-2 levels were produced by both strains at 0.995 a_W_ and 30 °C after 5 days of incubation. Overall, strain NRRL 66,749 produced more T-2 than strain NRRL 66,748 under the same conditions, 84.2 and 70.5 μg/g, respectively. T-2 production was higher at 30 °C, decreasing in the following order: 15 and 25 °C for both strains. At 25 °C, maximum amounts of T-2 were obtained at 0.995 a_W_ after 5 days of incubation for *F. chaquense* NRRL 66748, while for *F. chaquense* NRRL 66749, it was at 0.95 a_W_ after 15 days of incubation. At 15 °C, maximum amounts of T-2 were obtained at 0.98 a_W_ after 25 days of incubation for both *F. chaquense* strains.

The analysis of variance showed that T-2 production was significantly affected by a_W_, temperature x incubation time and a_W_ x incubation time interactions for *F. chaquense* NRRL 66748. While for *F. chaquense* NRRL 66749, a_W_, time of incubation and a_W_ x time of incubation interaction significantly influenced T-2 production, with a_W_ being the most important variable affecting T-2 production for both strains.

The highest production level of HT-2 was observed at 15 °C and 25 days of incubation for both strains, but at different a_W_: 0.95 and 0.98 for strain NRRL 66748 and NRRL 66749, respectively. Overall, strain NRRL 66,749 produced more HT-2 than strain NRRL 66748 under the same conditions, 25.6 and 12.8 μg/g, respectively. HT-2 production was higher at 15 °C, decreasing in the following order: 25 and 30 °C for both strains. At 30 °C, no toxin production was detected when a_W_ values were lower than 0.98 for any strain. At 25 °C, the maximum levels were obtained at 0.995 a_W_ after 15 days of incubation for both strains. While, at 15 °C, the maximum levels were obtained after 15 days of incubation, but at 0.95 a_W_ and 0.98 a_W_ for *F. chaquense* NRRL 66748 and NRRL 66749, respectively.

The analysis of variance of the HT-2 data showed that, for both strains, all the individual factors and some interactions significantly influenced the production of the toxin. a_W_ and incubation were the most important factors for NRRL 66748 and NRRL 66749 strains, respectively (Table 2).

Maximum BEA levels were produced by both strains at 0.995 a_W_ after 5 days of incubation, but at different temperatures: 25 °C and 30 °C for strain NRRL 66748 and NRRL 66749, respectively. Overall, both strains produced similar levels under the same conditions (7.4 and 6.9 μg/g). BEA production was higher at 30 °C, decreasing in the following order: 25 and 15 °C for strain NRRL 66749. Furthermore, for strain NRRL 66748, BEA production was higher at 25 °C, decreasing in the following order: 30 °C and 15 °C.

At the highest temperature assayed (30 °C), the strains differed in the a_W_ for maximum BEA production, being 0.98 and 0.995 after 5 days of incubation for the strain NRRL 66748 and NRRL 66749, respectively. At 25 °C, the maximum levels were obtained after 5 days of incubation, but at 0.995 a_W_ and 0.98 a_W_ for *F. chaquense* NRRL 66748 and NRRL 66749, respectively. At 15 °C, the maximum amount of BEA was observed at 0.98 a_W_ after 15 days of incubation for *F. chaquense* NRRL 66748. While for *F. chaquense* NRRL 66749, the maximum amount of BEA was observed at 0.995 after 5 days of incubation.

The analysis of variance indicated that, for both strains, BEA production was significantly affected by a_W_ and two- and three-way interactions (except temperatures x days of incubation by strain NRRL 66749). a_W_ was the most important factor for both strains (Table 2).

### 2.3. Effect of a_W_ and Temperature on Mycotoxin Profiles

**Figure 5:**
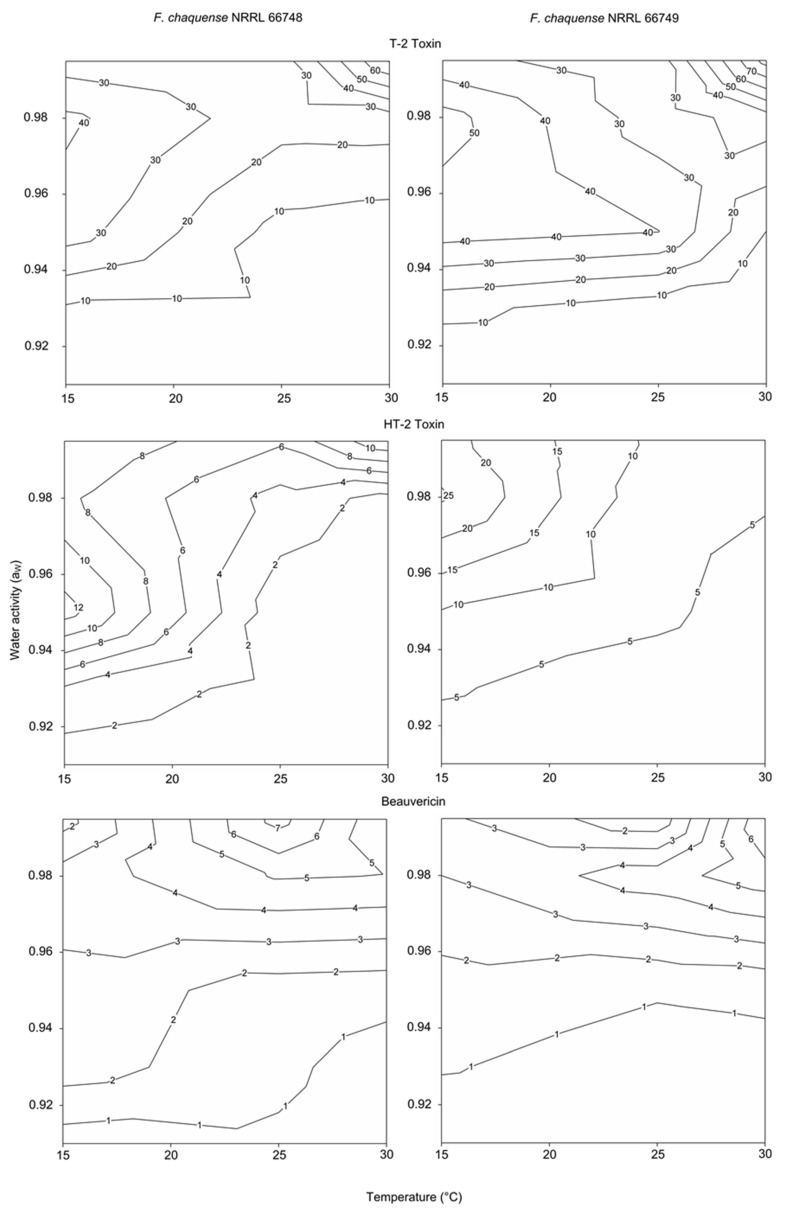
Two-dimensional contour maps of T-2, HT-2 and BEA production of *Fusarium chaquense* NRRL 66748 and NRRL 66749 in relation to temperature and water activity. The numbers on the isopleths refer to toxin levels (µg/g).

Figure 5 shows the two-dimensional contour maps obtained in order to identify the optimum conditions of a_W_, temperature and the range of conditions for the production of different amounts of mycotoxins by *F. chaquense*. Both strains of *F. chaquense* evaluated produced higher levels of T-2 at 0.995 a_W_ and 30 °C. Moreover, both strains produced appreciable levels of T-2 at 0.98 a_W_ and 15 °C. HT-2 showed the maximum level at 0.98–0.95 a_W_ and 15 °C. Finally, for BEA, maximum levels were obtained at 0.98–0.995 a_W_ and 25–30 °C.

## 3. Discussion

The FSAMSC represent one of the most taxonomically challenging groups of fusaria, comprising important mycotoxigenic plant pathogens as well as other species with various way of life. Among other toxins produced by members belonging to the FSAMSC, trichothecenes propose the most significant threat to public health [5]. Recently we described a novel member of the FSAMSC, *F. chaquense* isolated from asymptomatic native grasses (Poaceae) from a wetland ecosystem of the Chaco Province, Argentina. As part of this species characterisation, we conducted the present study in order to perform a deep analysis of the important type A trichothecene producer. From this study, it can be concluded that *F. chaquense* was able to grow in a grass-based media at almost all evaluated conditions: a_W_ values from 0.90 to 0.995, and three temperatures, 15, 25 and 30 °C. Lag phases results were similar to those previously discussed by Medina and Magan [15] in other *Fusarium* species.

Previous studies were performed on *Fusarium* species such as *F. langsethiae*, *F. sporotrichioides* and other species members of the FSAMSC, a lineage of *Fusarium* that produces trichothecene mycotoxins [16,17,18,19]. The results of the current study demonstrated that *F. chaquense* strains maximum growth ranged from 25 to 30 °C at 0.995 a_W_, reflecting the local climatic conditions of this species isolation region of Chaco wetlands. In this region, the annual temperature ranges between 20 and 24 °C. Maximum absolute temperatures can peak at 46.5 °C, and mean annual rainfall is 1300 mm, concentrated in spring and summer [20]. Growth was observed at the lowest assayed a_W_ (0.90) at all tested temperatures.

In previous studies, Kokkonen et al. [18] reported that species related to *F. chaquense*, such as *F. langsethiae* and *F. sporotrichoides*, were able to grow in an oat-based media with the maximal growth observed at 25 °C and 0.995 and 0.98 a_W_. Although, *F. sporotrichioides* can occur under a wider range of water stress conditions (a_W_ 0.95 and 0.93) than *F. langsethiae*. These results were similar to those reported by Medina and Magan [15], who studied the growth of *F. langsethiae* strains isolated from different northern European countries and also demonstrated that maximum growth was at 25 °C and 0.995 and 0.98 a_W_. In general, the present work results obtained were similar to those. Considering that *Fusarium* species can persist on a substrate during long periods of time, where a_W_ may change and temperature fluctuations may occur, the knowledge of optimal a_W_ and temperature range for growth is important. Moreover, in the field, the colonisation of developing grass by *F. chaquense* can be influenced by changes in relative humidity, temperature and rainfall.

Regarding mycotoxin production, in general, the maximum toxin levels were produced at different conditions than those optimal for *F. chaquense* growth. The mycotoxin production profile of both *F. chaquense* strains were in the order of importance: T-2, HT-2 and BEA. *Fusarium chaquense* mycotoxin production profile results similar to those previously described for *F. langsethiae* and *F. sporotrichioides* [21,22,23], which is the T-2 toxin, the type A trichothecene produced in the highest concentration by this species. However, the production of BEA by *F. langsethiae* and *F. sporotrichioides* has not been studied in depth. Our data show that trichothecenes synthesis predominates over BEA due to trichothecenes being detected in higher levels, at least under the assayed conditions of the present study.

The present study showed that the maximum T-2 level was produced by both strains at 0.995 a_W_ and 30 °C after 5 days of incubation, followed by at 0.98 a_W_ and 15 °C after 25 days of incubation. With regard to the other type A trichothecene studied, the highest level of HT-2 was obtained at 0.98–0.95 a_W_ and 15 °C after 25 days of incubation for both strains. Overall, strain NRRL 66749 produced higher levels of T-2 and HT-2 than the other strain under the same conditions. These results overlap with those obtained by Kokkonen et al. [18], who demonstrated that *F. sporotrichioides* strains produced more T-2 + HT-2 toxins at 15 °C and 0.995 a_W_, while *F. langsethiae* produced the maximum at 25 °C and 0.98 a_W_ in an oat-based media. Previous results of Kokkonen et al. [17] were obtained on a grain mixture where the conditions of 15 °C and 0.995 a_W_ favoured the toxin production by both *F. sporotrichioides* and *F. langsethiae*. Based on their findings, they concluded that a_W_ was the most important factor in controlling T-2 + HT-2 production, rather than temperature. As opposed to our study, the authors did not include 30 °C in their study because this temperature is not recorded in their country (Finland) during the cultivation of oats, wheat and barley, cereals in which *F. langsethiae* and *F. sporotrichioides* are responsible for the natural occurrence of type A trichothecenes. Medina and Magan [19] determined temperature and a_W_ effects on the production of T-2 and HT-2 by *F. langsethiae* strains from north European countries. Those authors included 30 and 35 °C in their study and demonstrated that the optimal conditions of a_W_ and temperature for T-2 were 0.98 and 20 °C, while for HT-2, they were 0.995 and 30 °C.

Regarding BEA production, the present study showed that the highest level was obtained at 0.995 a_W_ and 25–30 °C after 5 days of incubation for both strains. In general, the maximum BEA production levels by both strains were observed at similar incubation conditions; however, strains showed different behaviour. Kokkonen et al. [17] studied the effect of culture conditions on BEA production by seven *Fusarium* species on a grain mixture (wheat, oats and barley) at three different a_W_ and temperature combinations. The authors found that the maximum levels of this toxin for *F. sporotrichioides* occur at 0.96 a_W_ and 25 °C, and also that *F. poae* produced the maximum levels under two different conditions, 0.995 at 15 °C, and 0.96 at 25 °C. Recently, increased importance has been given to the investigation of this mycotoxin in cereals because of its toxic effects on plants and animals [24].

Data obtained from two-dimensional profiles of a_W_ by temperature interactions for *F. chaquense* allowed the recognition of areas where climate conditions could indicate a significant risk of grasses mycotoxin accumulation. It seems that mycotoxin production levels could be high not only when *F. chaquense* grows optimally but also when it grows under stress conditions. Moreover, it is important to remark the toxicological risk due to the possible interaction among the toxins detected because the simultaneous production of different toxic metabolites could imply additive and/or synergistic effects on target organisms. In addition, as *F. chaquense* is commonly associated with natural grasses in Argentina, the threat to animal health posed by this fungus should not be underestimated.

On the basis of our results, the climatic conditions that occur in the Chaco wetlands would be conducive for the development of *F. chaquense* and the production of mycotoxins. In addition, this species would appear to be very versatile, as it can grow and produce mycotoxins in a wide range of a_W_ and temperatures, which would give it an adaptive advantage over other *Fusarium* species and this would explain its high frequency of isolation in natural grasses growing in this wetland ecosystem.

## 4. Materials and Methods

### 4.1. Strains

Two *F. chaquense* strains, NRRL 66748 and NRRL 66749, isolated from asymptomatic plants belonging to the Poaceae family, collected from Chaco wetland, Argentina, during July 2011, were used in this study. These isolates were characterised by molecular and morphological criteria; whole genome sequence is also available [1]. The strains are preserved in the UNRC culture collection as spore suspensions in 15% glycerol frozen at −80 °C. Moreover, both strains were deposited in the Agricultural Research Service Culture Collection, Peoria, IL, USA (NRRL number).

### 4.2. Medium Preparation

Milled grasses were prepared by pulverising a mixture of *Paspalum* and *Panicum* in a mill with a 1 mm^2^ mesh (Cyclotech, Foss Tecator, Höganäs, Sweden). Mixtures of 2% (*w/v*) milled grass in water were prepared, and 2% (*w/v*) agar was added. The a_W_ of the basic medium was adjusted to 0.995, 0.98, 0.95, 0.93 and 0.91 by the addition of different amounts of glycerol [25]. The media were autoclaved at 120 °C for 20 min. Flasks of molten media were thoroughly shaken prior to pouring into 9 cm sterile Petri dishes. Using an Aqualab Series 3 (Decagon Devices, Inc., Pullman, WA, USA), the a_W_ of representative samples (2 of each treatment) of media was checked. Uninoculated control plates were also prepared and measured at the end of the experiment in order to detect any significant deviation of the a_W_.

### 4.3. Inoculation, Incubation and Growth Assessment

Petri plates were inoculated with a 3-mm-diameter agar disk taken from the margin of a 7-day-old colony of each isolate grown on synthetic nutrient agar [26] at 25 °C and transferred face down to the center of each plate. Inoculated plates of the same a_W_ were sealed in polyethylene bags and incubated at 15, 25 and 30 °C for 25 days. A full factorial design was used where the factors were a_W_, temperature and strain, and the response was growth (total number of plates: 5 a_W_ × 3 temperatures × 2 strains × 3 replicates).

For growth assessment, two diameters of the growing colonies were measured at right angles to each other every day for 25 days or until the colony reached the edge of the plate. Colonies radios were plotted against time, and linear regression was applied in order to obtain the growth rate (mm/day) as the slope of the line. At the end of the incubation period, uninoculated controls and treatments were frozen for later extraction and mycotoxins determination.

### 4.4. Mycotoxin Extraction

For mycotoxin extraction, all control and treatment Petri plates were used. Mycotoxins were extracted with 50 mL of methanol–water (90:10, *v/v*) by shaking half of the culture media (~10 g) and mycelia with the solvent for 60 min on an orbital shaker (150 rpm) and then filtering the extracts through a filter paper (No. 4; Whatman International Ltd., Maidstone, Kent, UK). An aliquot extract (1 mL) was transferred to an amber vial, evaporated to dryness at 50 °C under a moderate stream of nitrogen. Dry extracts were redissolved in 1 mL of acetonitrile: water (50:50, *v/v*) and preserved at −20 °C until HPLC analysis.

### 4.5. Mycotoxin Analysis

#### 4.5.1. Chemicals and Preparation of Standards

Mycotoxin standards (purity > 99%) were supplied by Sigma-Aldrich (Milan, Italy). All solvents (HPLC grade) were purchased from J. T. Baker (Deventer, The Netherlands). Water was of Milli-Q quality (Millipore, Bedford, MA, USA).

Mycotoxin stock solutions of T-2 and HT-2 toxins (1mg/mL each) were prepared by dissolving solid commercial toxins in acetonitrile (HPLC grade). A stock solution was prepared by mixing the simple toxin solutions and diluting them with acetonitrile into amber silanised vials to obtain a solution containing 20 μg/mL of each toxin. Aliquots of the stock solution were evaporated to dryness under a stream of nitrogen at 50 °C. The residue was dissolved with water/acetonitrile (80:20, *v/v*) to obtain calibrated standard solutions at 0.05, 0.10, 0.20, 0.40, 1.00, 2.00 and 4.00 µg/mL of T-2 and HT-2 toxins.

Standards stock solution of BEA (1 mg/mL) were prepared by dissolving the solid commercial toxin standards in methanol into amber silanised vials to obtain a solution containing 100 μg/mL of toxin. Adequate amounts of the stock solution were dried under a nitrogen stream at 50 °C and reconstituted with methanol/water (70:30, *v/v*) to obtain calibrant standard solutions since 0.02 a 40.00 µg/mL. Standard solutions were stored at -20 °C and warmed after.

#### 4.5.2. Determination of Type A Trichothecenes (T-2 and HT-2)

Type A Trichothecenes were detected using the method previously described by Pascale et al. [27]. T-2 and HT-2 toxins analysis was performed using a UHPLC (Agilent UHPLC system, 1290 Series). Both data acquisition and instrument control were performed by LC Openlab software (Agilent). For chromatographic separation, a reversed-phase column of C_18_ (50 × 2.1 mm i.d., 1.8 μm, ZORBAX Eclipse Plus) was used. Analyses were performed in the gradient mode. Solvent A was water and solvent B acetonitrile. Gradient conditions were initiated by holding for the first 1.5 min with 30% B, and then solvent B was linearly increased to 35% in 0.5 min and kept constant for 2 min. The flow rate was 0.5 mL/min, and the injection volume was 10 μL. The column temperature was maintained at 50 °C, and the detector was set at 202 nm wavelength. Retention times were 1.97 min and 4.9 min for HT-2 and T-2, respectively. The mycotoxins were quantified by comparing peak areas with calibration curves obtained with standard solutions. The detection limit (LOD) based on a signal-to-noise ratio of 3:1 for both toxins was 0.24 μg/g.

#### 4.5.3. Determination of Beauvericin

Beauvericin was detected using the method previously described by Prosperini et al. [28]. BEA analysis was performed using an HPLC (Agilent 1260 Series, Agilent Technology, Santa Clara, CA, USA) equipped with a binary solvent manager and a diode array (DAD). Both data acquisition and instrument control were performed by LC Openlab software (Agilent). For chromatographic separation, a reversed-phase column of C_18_ (150 × 4.6 mm i.d., 5 μm, Gemini-Phenomenex, Torrance, CA, USA) with a guard column SecurityGuard™ (4 × 3.0 mm) of the same material was used. Analyses were performed in the gradient mode. Solvent A was water and solvent B acetonitrile. Gradient conditions were initiated by holding for the first 5 min with 70% B, then solvent B was linearly increased to 90% in 10 min and kept constant for 1 min. The column was re-equilibrated with 70% eluent B for 4 min. The flow rate was 1.0 mL/min, and the injection volume was 100 μL. The column temperature was maintained at 40 °C, and the detector was set at 205 nm wavelength, with a retention time of 11.4 min. The mycotoxin was quantified by comparing peak areas with calibration curves obtained with standard solutions. The detection limit (LOD), based on a signal-to-noise ratio of 3:1, was 0.04 μg/g.

A recovery experiment was performed in triplicate by spiking of the final working solution to obtain levels of 500 ng/mL of T-2, HT-2 and BEA 2% milled grass agar culture medium, previously sterilised and cooled to approximately 50 °C, then it was homogenised by shaking and distributed at a rate of 20 mL per Petri dish. Once the culture medium had solidified, the toxins were extracted, detected and quantified following the methodologies described above. The mean recovery was 99%, 98% and 98% for T-2, HT-2 and BEA, respectively.

### 4.6. Statistical Analysis

The growth rate, lag phase and mycotoxin concentration were evaluated by analysis of variance (ANOVA) using InfoStat version 2016 [29]. Statistical significance was determined at *p* < 0.01.

## Figures and Tables

**Table 1:** Analysis of variance on the effect of water activity (a_W_), temperature (T) and different strains (S) and their interactions on growth rates and lag phases of *Fusarium chaquense* grown in a grass-based media.

Source of Variation	Df ^a^	Lag Phase (h)		Growth Rate (mm/day)	
		MS ^b^	F ^c^	MS	F
S	1	0.01	31.85 *	30.66	831.09 *
T	2	0.11	241.97 *	0.09	23.63 *
a_W_	4	0.14	307.61 *	0.63	168.57 *
S × T	2	0.01	19.79 *	1.08	288.61 *
S × a_W_	4	0.01	15.19 *	13.41	3585.19 *
T × a_W_	8	0.04	98.41 *	0.03	7.42 *
S × T × a_W_	8	0.01	17.38 *	0.10	25.91 *

* Significant *p* < 0.01. ^a^ Degrees of freedom. ^b^ Mean square. ^c^ Snedecor-F.

**Table 2:** Analysis of variance on the effects of water activity (a_W_), temperature (T), incubation time (D) and their interactions on three mycotoxin production by *Fusarium chaquense* strains grown in a grass-based media.

**NRRL 66748**							
**Source of variation**	**Df ^a^**	**T-2**		**HT-2**		**BEA**	
		**MS ^b^**	**F ^c^**	**MS**	**F**	**MS**	**F**
T	2	188.37	1.32	59.04	14.62 *	0.01	0.12
a_W_	4	2938.12	20.64 *	72.81	18.03 *	3.14	31.28 *
D	2	220.05	1.55	24.29	6.01 *	0.12	1.22
T × a_W_	8	363.2	2.55	10.57	2.62 *	0.44	4.36 *
T × D	4	722.38	5.07 *	41.1	10.18 *	0.66	6.52 *
a_W_ × D	8	558.43	3.92 *	6.38	1.58	0.33	3.24 *
T × a_W_ × D	16	228.5	1.61	11.12	2.75 *	0.24	2.41 *
**NRRL 66749**							
**Source of variation**	**Df ^a^**	**T-2**		**HT-2**		**BEA**	
		**MS ^b^**	**F ^c^**	**MS**	**F**	**MS**	**F**
T	2	1.02	7.26	9.13	30.46 *	0.3	2.73
a_W_	4	7.03	49.84 *	7.6	25.37	3.48	31.46 *
D	2	1.21	8.54 *	14.96	49.91 *	0.2	1.85
T × a_W_	8	0.3	2.11	0.86	2.87	0.4	3.60 *
T × D	4	0.4	2.82	2.56	8.55	0.2	1.85
a_W_ × D	8	0.71	5.05 *	1.97	6.59	0.3	2.75 *
T × a_W_ × D	16	0.12	0.88	0.57	1.9	0.25	2.25 *

* Significant *p* < 0.01. ^a^ Degrees of freedom. ^b^ Mean square. ^c^ Snedecor-F.

## References

[1] Nichea M., Proctor R., Probyn C., Palacios S., Cendoya E., Sulyok M., Chulze S., Torres A., Ramirez M. (2021). *Fusarium chaquense* sp. nov, a novel type A trichothecene-producing species from native grasses in a wetland ecosystem in Argentina. Mycologia.

[2] Yli-mattila T., Ward T.J., Donnell K.O., Proctor R.H., Burkin A.A., Kononenko G.P., Gavrilova O.P., Aoki T., McCormick S.P., Yu T. (2011). *Fusarium sibiricum* sp. nov., a novel type A trichothecene-producing *Fusarium* from northern Asia closely related to *F. sporotrichioides* and *F. langsethiae*. Int. J. Food Microbiol..

[3] Rocha L.O., Laurence M.H., Proctor R.H., Mccormick S.P., Summerell B.A., Liew E.C.Y. (2015). Variation in type A trichothecene production and trichothecene biosynthetic genes in *Fusarium goolgardi* from natural ecosystems of Australia. Toxins.

[4] Nichea M., Palacios S., Chiacchiera S., Sulyok M., Krska R., Chulze S., Torres A., Ramirez M. (2015). Presence of multiple mycotoxins and other fungal metabolites in native grasses from a wetland ecosystem in Argentina intended for grazing cattle. Toxins.

[5] Laraba I., McCormick S.P., Vaughan M.M., Geiser D.M., O’Donnell K. (2021). Phylogenetic diversity, trichothecene potential, and pathogenicity within *Fusarium sambucinum* species complex. PLoS ONE.

[6] Agriopoulou S., Stamatelopoulou E., Varzakas T. (2020). Advances in occurrence, importance, and mycotoxin control strategies: Prevention and detoxification in foods. Foods.

[7] Munkvold G.P., Moretti A., Susca A. (2017). Fusarium species and their associated mycotoxins. Mycotoxigenic Fungi: Methods and Protocols.

[8] Proctor R.H., McCormick S.P., Gutiérrez S. (2020). Genetic bases for variation in structure and biological activity of trichothecene toxins produced by diverse fungi. Appl. Microbiol. Biotechnol..

[9] McCormick S.P., Stanley A.M., Stover N.A., Alexander N.J. (2011). Trichothecenes: From simple to complex mycotoxins. Toxins.

[10] Charmley L.L., Rosenberg A., Trenholm H.L., Miller J.D., Trenholm H.L. (1994). Factors responsible for economic losses due to *Fusarium* mycotoxin contamination of grains, foods and feedstuffs. Mycotoxins in Grains: Compounds Other than Aflatoxin.

[11] Marin S., Magan N., Ramos A.J., Sanchis V. (2004). Fumonisin-producing strains of *Fusarium*: A review of their ecophysiology. J. Food Prot..

[12] Leslie J.F., Zeller K.A., Logrieco A., Mule G., Moretti A., Ritieni A. (2004). Species diversity of and toxin production by *Gibberella fujikuroi* species complex strains isolated from native prairie grasses in Kansas. Appl. Environ. Microbiol..

[13] Phan H.T., Burgess L.W., Summerell B.A., Bullock S., Liew E.C.Y., Clarkson J.R. (2004). *Gibberella gaditjirrii* (*Fusarium gaditjirrii*) sp. nov., a new species from tropical grasses in Australia. Stud. Mycol..

[14] Sanchez Marquez S., Bills G.F., García Criado B., Zabalgogeazcoa I. (2008). Diversity and structure of the fungal endophytic assemblages from two sympatric coastal grasses. Fungal. Divers..

[15] Medina A., Magan N. (2010). Comparisons of water activity and temperature impacts on growth of *Fusarium langsethiae* strains from northern Europe on oat-based media. Int. J. Food Microbiol..

[16] Mateo J.J., Mateo R., Jime M. (2002). Accumulation of type A trichothecenes in maize, wheat and rice by *Fusarium sporotrichioides* isolates under diverse culture conditions. Int. J. Food Microbiol..

[17] Kokkonen M., Ojala L., Parikka P., Jestoi M. (2010). Mycotoxin production of selected *Fusarium* species at different culture conditions. Int. J. Food Microbiol..

[18] Kokkonen M., Jestoi M., Laitila A. (2012). Mycotoxin production of *Fusarium langsethiae* and *Fusarium sporotrichioides* on cereal-based substrates. Micotoxin Res..

[19] Medina A., Magan N. (2011). Temperature and water activity effects on production of T-2 and HT-2 by *Fusarium langsethiae* strains from north European countries. Food Microbiol..

[20] Alberto J.A. (2006). El Chaco oriental y sus fisonomías vegetales. Geográfica Digit..

[21] Langseth W. (1998). Mycotoxin production and cytotoxicity of *Fusarium* strains isolated from Norwegian cereals. Mycopathologia.

[22] Thrane U., Adler A., Clasen P.E., Galvano F., Langseth W., Lew H., Logrieco A., Nielsen K.F., Ritieni A. (2004). Diversity in metabolite production by *Fusarium langsethiae*, *Fusarium poae*, and *Fusarium sporotrichioides*. Int. J. Food Microbiol..

[23] Jestoi M.N., Paavanen-Huhtala S., Parikka P., Yli-Mattila T. (2008). *In vitro* and *in vivo* mycotoxin production of *Fusarium* species isolated from Finnish grains. Arch Phytopathol. Pflanzenschutz.

[24] Jestoi M. (2008). Emerging *Fusarium*-mycotoxins fusaproliferin, beauvericin, enniatins, and moniliformin-A review. Crit. Rev. Food Sci. Nut..

[25] Dallyn H., Fox A., Gould G.H., Corry J.E.L. (1980). Spoilage of materials of reduced water activity by xerophilic fungi. Microbial Growth and Survival in Extremes of Environment.

[26] Gerlach W., Nirenberg H. (1982). The Genus Fusarium—A Pictorial Atlas.

[27] Pascale M., Panzarini G., Visconti A. (2012). Determination of HT-2 and T-2 toxins in oats and wheat by ultra-performance liquid chromatography with photodiode array detection. Talanta.

[28] Prosperini A., Meca G., Font G., Ruiz M.J. (2012). Study of the cytotoxic activity of beauvericin and fusaproliferin and bioavailability in vitro on Caco-2 cells. Food Chem. Toxicol..

[29] Di Rienzo J.A., Casanoves F., Balzarini M., Gonzalez L., Cuadroda M., Robledo C. (2018). InfoStat Versión 2016.

